# Metastatic Infiltrating Ductal Carcinoma of the Breast to the Colon: A Case Report and Literature Review

**DOI:** 10.1155/2013/603683

**Published:** 2013-10-01

**Authors:** Salih Samo, Muhammed Sherid, Husein Husein, Samian Sulaiman, Jeffrey V. Brower, Seth Alper, Margaret Yungbluth, John A. Vainder

**Affiliations:** ^1^Division of Gastroenterology, Department of Internal Medicine, Saint Francis Hospital Evanston, Program, University of Illinois at Chicago, 355 Ridge Avenue, Evanston, IL 60202, USA; ^2^Division of Gastroenterology, Department of Internal Medicine, CGH Medical Center, 100 East LeFevre Road, Sterling, IL 61081, USA; ^3^Division of Gastroenterology, Department of Internal Medicine, University of Tishreen, Aleppo Street, P.O. Box 2230, Latakia, Syria; ^4^Department of Radiology, Saint Francis Hospital Evanston Program, University of Illinois at Chicago, 355 Ridge Avenue, Evanston, IL 60202, USA; ^5^Department of Pathology, Saint Francis Hospital Evanston Program, University of Illinois at Chicago, 355 Ridge Avenue, Evanston, IL 60202, USA

## Abstract

True metastatic involvement of the colon is rare. Colonic metastases occur most commonly secondary to peritoneal metastases from intra-abdominal malignancies. Breast cancer is the most common malignancy that metastasizes hematogenously to the colon. Colonic metastatic disease mimics primary colonic tumors in its presentation. Colonic metastatic involvement is a poor prognostic sign, and the pathologist should be informed about the history of the primary breast cancer when examining the pathologic specimens. In this paper, we report a case of an ileocecal mass found to be histologically consistent with metastatic ductal breast cancer, and then we review the literature about breast cancer metastases to the gastrointestinal tract in general and colon in particular.

## 1. Introduction

Metastatic involvement of the gastrointestinal (GI) tract is generally an infrequent clinical entity [1–3]. Colorectal involvement is particularly rare in the setting of metastatic diseases [4, 5] as opposed to small bowel, where metastatic disease is more common than primary malignancies [4]. Indeed, small bowel is the most common location of metastases within the GI tract, followed by the stomach [6, 7] owing to the rich vascular supply of these two locations [8]. Variability in the prevalence of metastatic involvement of these two common sites of metastasis is, however, observed with different primaries. Interestingly, in the setting of metastatic breast cancer, the stomach is the most frequent site within the GI tract for metastases and usually presents as linitis plastica [8–10]. Colonic metastases are much less common in the clinical setting of metastatic breast cancer.

Metastatic involvement of the colon occurs most commonly secondary to peritoneal seeding from intra-abdominal malignancies. The most frequent location in which seeding occurs is in the pouch of Douglas [5, 11, 12]. Therefore, colonic involvement from surrounding intra-abdominal tumors (e.g., ovarian carcinoma) is much more common than hematogenous seeding [5]. Colorectal metastases can occur via various different pathways. Pelvic neoplasms can spread by direct invasion through the fasciae and mesenteric attachments or, more commonly, through the mesenteric reflections. The mesosigmoid and the right paracolic gutters are less commonly involved [11, 12].

Peritoneal carcinomatosis may arise from different locations: intra-abdominal primary tumors (e.g., mesothelioma) and intra-abdominal viscera (e.g., colon adenocarcinoma and ovarian carcinoma), or they may spread from extra-abdominal malignancies (e.g., breast cancer and melanoma).

Here, we report a case of an ileocecal mass found to be histologically consistent with metastatic ductal breast cancer 4 years from the initial diagnosis of the primary breast malignancy. 

## 2. Case Information

A 76-year-old female presented to the emergency room with a one-day history of shortness of breath and chest tightness. She denied having fever, chills, palpitations, or cough. She endorsed a history of estrogen receptors (ER) positive breast cancer with known metastases involving the bones 4 years prior to presentation, atrial fibrillation, and hypertension. The patient received adjuvant radiation therapy for her metastatic breast cancer after undergoing left mastectomy and was put on estrogen antagonist hormonal therapy at the time of diagnosis.

On physical examination, the patient was afebrile with stable vital signs. She was pale in appearance. Cardiopulmonary examination was unremarkable, and her abdominal examination revealed no abdominal tenderness, distention, or masses. Fecal occult blood was positive with no masses palpated in the rectal vault.

**Figure 1:**
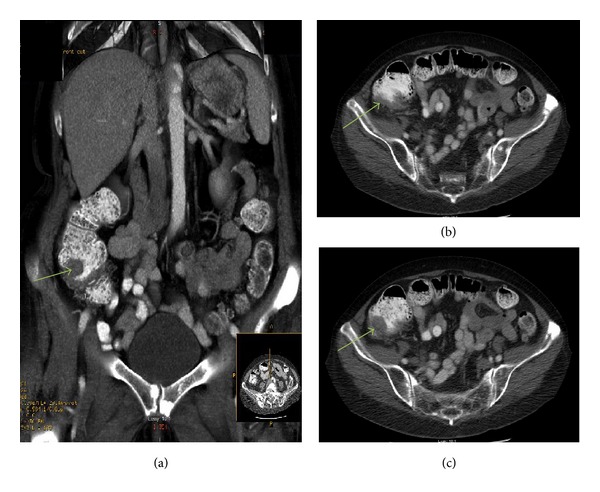
CT abdomen and pelvis. (a) (coronal view) and (b) and (c) (axial views) show an area of irregular wall thickening in the cecum (green arrows).

Laboratory studies were remarkable only for a hemoglobin of 8.2 g/dL (normal 12–15.3 g/dL) and hematocrit of 25.8% (normal 34.7–45.1%) with an MCV of 88.7 fl (normal 80–100 fl). Computed tomography (CT) scan of the abdomen and pelvis showed irregular wall thickening in the cecum (Figure 1). During colonoscopy and while trying to pass the scope through the angulated bowel the patient started suddenly bleeding from the sigmoid colon with some fat being seen, so the procedure was aborted. Following colonoscopy, the patient began to report generalized abdominal pain. Abdominal X-ray subsequently demonstrated intra-abdominal free air. 

The patient was emergently taken to the operation room where she was found to have a perforation in the distal sigmoid colon and a 2 × 3 cm mass at the ileocecal valve, with peritoneal studding. Limited resection was performed via ileocecectomy with closure of the perforation.

**Figure 2:**
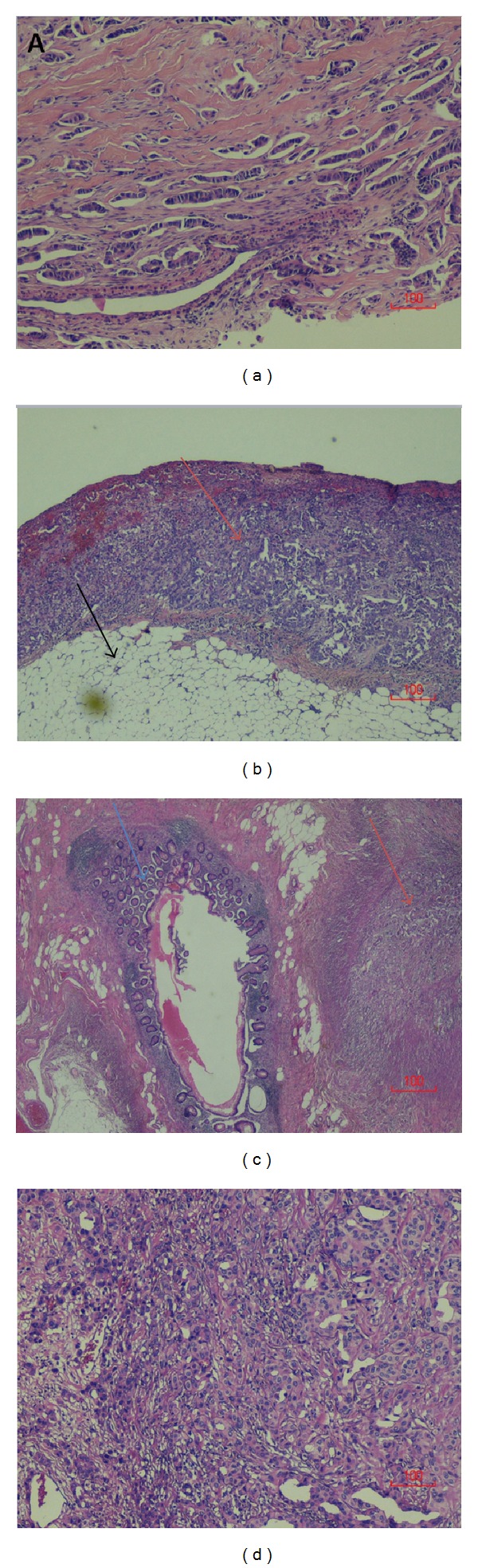
(a) Core needle biopsy of the left breast shows an invasive, moderately differentiated infiltrating ductal cell carcinoma with microvascular invasion (H&E, 100x). (b) An invasive, poorly differentiated carcinoma (red arrows) involving the intra-abdominal adipose tissue (black arrow) (H&E, 40x) and (c) the serosal aspect of the appendix (blue arrow = appendiceal mucosa; red arrow = serosal tumor) (H&E, 20x) consistent with breast cancer. (d) Tumor in appendiceal serosa (H&E, 100x).

**Figure 3:**
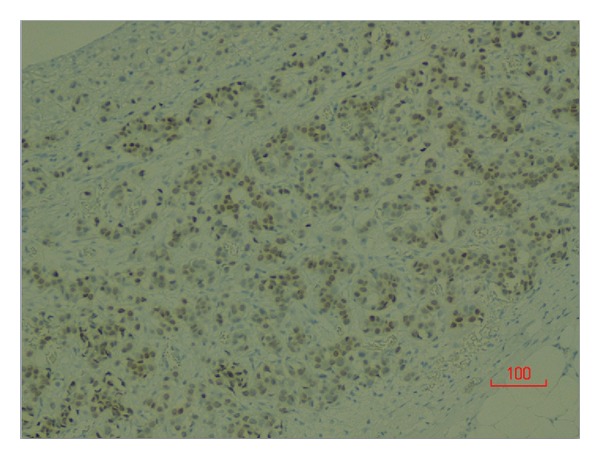
ER positive tumor cells invading the intra-abdominal adipose tissue.

Histopathologic examination from samples retrieved during the procedure was consistent with an invasive poorly differentiated ER positive carcinoma involving the cecum and appendix (Figures 2(c) and 2(d)) and the intra-abdominal adipose tissue (Figures 2(b) and 3), consistent with a breast cancer as a primary tumor. Intra-abdominal lymph node samplings were also consistent with a metastatic disease from breast cancer. Adjuvant chemotherapy with cyclophosphamide and doxorubicin was administered postoperatively, following surgical recovery.

## 3. Discussion

Malignant melanoma was thought to be the most common tumor metastasizing hematogenously to the colon [13]. However, a recent study by Mourra et al., in which thirty-five patients with confirmed metastatic colorectal involvement out of 10,365 patients with colorectal malignancies were found, reported that breast carcinoma (17 cases) was the leading cause of colonic metastases followed by melanoma (7 cases), lung carcinoma, and sarcoma (4 cases each) [5]. Another study conducted on 16,000 autopsies over a duration of 10 years discovered only 62 cases of colonic metastases with the most common primaries being lung (14 cases) and breast (10 cases) [14].

Among breast cancer subtypes, infiltrating lobular carcinoma was found to metastasize more frequently to the GI tract in a study of 2,604 patients with breast cancer over a period of 18 years [6]. Only 17 cases (<1%) were found to metastasize to the GI tract [6]. Further, lobular breast carcinoma tends to metastasize to various locations including the GI tract, gynecological organs, peritoneum, and retroperitoneum, whereas ductal carcinoma tends to metastasize more frequently to the brain, lung, and liver [15]. The reason for this dissemination pattern observed in the setting of lobular carcinoma has been linked to a particular tropism of the metastatic cells of lobular ductal carcinoma [2, 16]. Also, lobular carcinoma has the tendency to cause more diffuse GI tract disease as opposed to ductal carcinoma which tends to cause more nodular lesions [1]. The interval from the initial diagnosis of breast cancer to the presentation of GI metastases has been reported for up to 26 years [17, 18].

Metastases to the lower GI tract can present with various nonspecific symptoms similar to those of primary colorectal tumors. Presenting symptoms often include abdominal pain, anorexia, nausea, vomiting, change in bowel habits, bleeding, obstruction, or, less frequently, perforation [5, 11, 12, 19–24]. The nonspecific presentation often makes it very difficult to differentiate primary colorectal tumors from breast cancer metastases, which usually occur several years after the initial diagnosis of breast cancer [7]. Clinically, primary colorectal tumors are indistinguishable from secondary ones without histological examination [5].

CT scan is a useful tool for the noninvasive assessment of degree of tumor spread within the bowel wall [9]. With the new multiphase, multidetector CT scans, the accuracy of assessing mural penetration of metastases into the bowel wall is very high [25, 26]. Ultrasonographic examination has been shown to be a useful imaging modality in several reports.

Although endoscopy plays an indispensable role in the diagnosis of GI tract metastases [27], the endoscopic features may not distinguish primary colon cancer from metastatic one; however, the majority of breast cancer metastases to the colon present as diffuse thickening of the colonic wall which is easily identified via direct visualization with colonoscopy [7]. Metastatic breast lesions involving the colonic mucosa may also present as ulcerated or nodular lesions resembling inflammatory bowel disease [28]. During colonoscopic examination, if the lesions are sufficiently deep, care must be taken to avoid superficial biopsies which might result in false negative histopathological examination [7].

Absence of colonic epithelial dysplasia is an important key to differentiate the primary colonic lesions from the secondary colonic lesions [29]; however, the finding of “signet ring” neoplasia, which can be found in some ductal breast carcinomas, can be confusing as it usually suggests the GI tract as the primary origin of the tumor [27]. The “Indian file” histological finding has been described when the infiltrating tumor cells cause a severe fibrous reaction leading to the linitis plastica appearance [30, 31].

Surgical management has not proven to result in an overall survival benefit except in selected cases where metastases are limited to a discrete region of the GI tract [6]. A retrospective analysis by McLemore et al. demonstrated that surgical intervention did not have significant survival benefits for patients with GI tract metastases from breast cancer [16]. Since colorectal metastases indicate a poor prognosis and usually represent the late stage of the disease [5], management in general is hormonal therapy or chemotherapy with surgery being mainly indicated for palliative purposes and symptomatic treatment (e.g., obstruction or bleeding) [32].

In the study by Mourra et al., none of the patients survived 5 years after the diagnosis of colorectal metastases. The mean survival time was longer by only 3 months in patients with surgical treatment than in those without surgery (12.35 versus 9.8 months) [5]. However, the number of patients in this series (especially those without surgery) is too small to draw a conclusion.

In our case, it is very difficult to determine whether the colonic tumor was an extension from the peritoneal carcinomatosis or from hematogenous metastases. However, it is very possible that the tumor was an extension from the peritoneal carcinomatosis since the tumor was recognized on the serosal surface of the appendix. On the other side, intra-abdominal lymph nodes involvement may argue against this possibility. 

In conclusion, metastatic breast cancer involving the GI tract is a poor prognostic indicator. Imaging studies, endoscopy, and histopathology are essential for the prompt and definitive diagnosis. Despite the paucity of true colonic metastases, the pathologist should be made aware of the history of breast cancer and the possibility of metastatic disease when examining any colonic biopsy. Our case represents an unusual metastatic pattern of breast ductal cell carcinoma to the GI tract.

## References

[1] Pectasides D, Psyrri A, Pliarchopoulou K (2009). Gastric metastases originating from breast cancer: report of 8 cases and review of the literature. *Anticancer Research*.

[2] Nazareno J, Taves D, Preiksaitis HG (2006). Metastatic breast cancer to the gastrointestinal tract: a case series and review of the literature. *World Journal of Gastroenterology*.

[3] Taal BG, Peterse H, Boot H (2000). Clinical presentation, endoscopic features, and treatment of gastric metastases from breast carcinoma. *Cancer*.

[4] Iacobuzio-Donahue C, Groisman GM, Bosman FT, Carnieiro F, Hruban RH, Theise ND (2010). Secondary tumours of the small intestine. *WHO Classification of Tumours of the Digestive System*.

[5] Mourra N, Jouret-Mourin A, Lazure T (2012). Metastatic tumors to the colon and rectum: a multiinstitutional study. *Archives of Pathology & Laboratory Medicine*.

[6] Borst MJ, Ingold JA, Hasselgren PO (1993). Metastatic patterns of invasive lobular versus invasive ductal carcinoma of the breast. *Surgery*.

[7] Nikkar-Esfahani A, Kumar BG, Aitken D, Robert Wilson G (2013). Metastatic breast carcinoma presenting as a sigmoid stricture: report of a case and review of the literature. *Case Reports in Gastroenterology*.

[8] So YK, Kyoung WK, Ah YK (2006). Bloodborne metastatic tumors to the gastrointestinal tract: CT findings with clinicopathologic correlation. *American Journal of Roentgenology*.

[9] Ambroggi M, Stroppa EM, Mordenti P (2012). Metastatic breast cancer to the gastrointestinal tract: report of five cases and review of the literature. *International Journal of Breast Cancer*.

[10] Cormier WJ, Gaffey TA, Welch JM (1980). Linitis plastica caused by metastatic lobular carcinoma of the breast. *Mayo Clinic Proceedings*.

[11] Caramella E, Bruneton JN, Roux P (1983). Metastases of the digestive tract. Report of 77 cases and review of the literature. *European Journal of Radiology*.

[12] Feczko PJ, Collins DD, Mezwa DG (1993). Metastatic disease involving the gastrointestinal tract. *Radiologic Clinics of North America*.

[13] Samo S, Sherid M, Husein H, Sulaiman S, Vainder JA (2013). Metastatic malignant melanoma to the colon: a case report and review of the literature. *Journal of Gastrointestinal Cancer*.

[14] Berge T, Lundberg S (1977). Cancer in Malmö 1958–1969. An autopsy study. *Acta Pathologica et Microbiologica Scandinavica*.

[15] Fondrimer E, Guérin O, Lorimier G (1997). A comparative study of metastatic patterns of ductal and lobular carcinoma of the breast from two matched series (376 patients). *Bulletin du Cancer*.

[16] McLemore EC, Pockaj BA, Reynolds C (2005). Breast cancer: presentation and intervention in women with gastrointestinal metastasis and carcinomatosis. *Annals of Surgical Oncology*.

[17] Haberstich R, Tuech JJ, Wilt M, Rodier JF (2005). Anal localization as first manifestation of metastatic ductal breast carcinoma. *Techniques in Coloproctology*.

[18] Théraux J, Bretagnol F, Guedj N, Cazals-Hatem D, Panis Y (2009). Colorectal breast carcinoma metastasis diagnosed as an obstructive colonic primary tumor. A case report and review of the literature. *Gastroenterologie Clinique et Biologique*.

[19] Rees BI, Okwonga W, Jenkins IL (1976). Intestinal metastases from carcinoma of the breast. *Clinical Oncology*.

[20] Graham AP (1947). Malignancy of the kidney: survey of 195 cases. *Journal of Urology*.

[21] Cifuentes N, Pickren JW (1979). Metastases from carcinoma of mammary gland: an autopsy study. *Journal of Surgical Oncology*.

[22] Taal BG, Den Hartog Jager FCA, Steinmetz R, Peterse H (1992). The spectrum of gastrointestinal metastases of breast carcinoma. II: the colon and rectum. *Gastrointestinal Endoscopy*.

[23] Graham WP, Goldman L (1964). Gastrointestinal metastases from carcinoma of the breast. *Annals of Surgery*.

[24] Harris M, Howell A, Chrissohou M (1984). A comparison of the metastatic pattern of infiltrating lobular carcinoma and infiltrating duct carcinoma of the breast. *British Journal of Cancer*.

[25] Uzzaman MM, Alam A, Nair MS, Borgstein R, Meleagros L (2012). Computed tomography findings of bowel wall thickening: its significance and relationship to endoscopic abnormalities. *Annals of the Royal College of Surgeons of England*.

[26] Wei WZ, Yu JP, Li J, Liu CS, Zheng XH (2005). Evaluation of contrast-enhanced helical hydro-CT in staging gastric cancer. *World Journal of Gastroenterology*.

[27] Bamias A, Baltayiannis G, Kamina S (2001). Rectal metastases from lobular carcinoma of the breast: report of a case and literature review. *Annals of Oncology*.

[28] Koos L, Field RE (1980). Metastatic carcinoma of breast simulating Crohn’s disease. *International Surgery*.

[29] Franceschini G, Manno A, Mulè A (2006). Gastro-intestinal symptoms as clinical manifestation of peritoneal and retroperitoneal spread of an invasive lobular breast cancer: report of a case and review of the literature. *BMC Cancer*.

[30] Cervi G, Vettoretto N, Vinco A (2001). Rectal localization of metastatic lobular breast cancer report of a case. *Diseases of the Colon and Rectum*.

[31] Tavassoli FA (1999). Infiltrating carcinoma: common and familiar special types. *Pathology of the Breast*.

[32] Titi MA, Anabtawi A, Newland AD (2010). Isolated gastrointestinal metastasis of breast carcinoma: a case report. *Case Reports in Medicine*.

